# Decoding G-Quadruplexes Sequence in *Vitis vinifera*: Regulatory Region Enrichment, Drought Stress Adaptation, and Sugar–Acid Metabolism Modulation

**DOI:** 10.3390/plants14081180

**Published:** 2025-04-10

**Authors:** Jun Xie, Kangkang Song, Gaixia Qiao, Rong Wang, Hongyuan Wu, Qiaoxia Jia, Yujuan Liu, Yi Li, Meilong Xu

**Affiliations:** 1 College of Forestry, Gansu Agriculture University, Lanzhou 730070, China; xiejun9188@163.com; 2 State Key Laboratory of Efficient Production of Forest Resources, Yinchuan 750004, China; 3 College of Forestry, Shandong Agricultural University, Tai’an 271018, China; kangkangsong234@163.com; 4 Beijing Anling Ecological Construction Co., Ltd., Beijing 102300, China; 5 Horticulture Research Institute, Ningxia Academy of Agricultural and Forestry Sciences, Yinchuan 750012, China

**Keywords:** *Vitis vinifera*, G-quadruplex, transcriptional regulation, drought stress, sugar–acid metabolism

## Abstract

G-quadruplexes play a crucial role in transcription, translation, and DNA replication in plant genomes. Here, we comprehensively examined the prevalence and functions of G-quadruplexes in *Vitis vinifera*. A total of 467,813 G-quadruplexes were identified in grapevine genome, with enrichment in the promoter (0.54/kbp) and near transcription start sites (TSSs, 1.00/kbp), and showed conservative strand preference. The G-quadruplex density in centromeres exhibited heterogeneity. The differentially expressed genes (DEGs) under two-day drought stress manifested high G-quadruplex density in the promoter and TSS regions. The upregulated DEGs showed template strand-biased G-quadruplex enrichment, while downregulated DEGs displayed coding strand dominance linked to metal ion homeostasis and sugar–acid metabolism pathways, respectively. G-quadruplexes were enriched in key sugar–acid metabolism genes, including pyruvate kinase and sucrose synthase. The number of G-quadruplexes in sucrose transferase VINV genes was higher than that in the CWINV and NINV genes. This study revealed G-quadruplexes as regulatory elements of stress response and berry development, providing abundant genetic targets for precision breeding and the quality improvement of grapevines.

## 1. Introduction

The G-quadruplexes are novel nucleic acid secondary structures formed by the folding of guanine-rich DNA or RNA sequences [1,2]. The fundamental structural unit is the G-tetrad, where four guanine bases are paired through Hoogsteen hydrogen bonding [3]. Cations (such as K^+^, Na^+^, Ca^2+^, Mg^2+^) could coordinate with the carbonyl oxygen atoms within the G-tetrad, thereby enhancing the stability of the G-quadruplex [4]. These structures are widely distributed in eukaryotic genomes and are particularly enriched in telomeres, promoters, enhancers, and non-coding regions [5,6]. In the promoter regions of cancer-related genes (such as *MYC*, *KRAS*, and *VEGF*), the formation of G-quadruplexes regulates the suppression or activation of oncogenes [7,8,9,10]. Numerous functional studies have demonstrated that G-quadruplexes play crucial roles in key biological processes such as gene transcription, DNA replication, genomic stability, and epigenetic regulation by modulating chromatin accessibility, impeding replication fork progression, or recruiting transcription factors [11,12]. G-quadruplexes have been extensively studied in humans. However, the characteristics and functions of G-quadruplexes in plant genomes remain largely unexplored.

Studies focusing on G-quadruplexes in plant genomes have been gradually expanded [13,14,15]. In *Arabidopsis thaliana*, genome-wide prediction discovered 43,000 G-quadruplexes, 80% of which were located in the gene region [16]. The enrichment of G-quadruplexes has also been observed in gene regions, promoters, and UTR regions in rice [17]. In *Spirodela polyrhiza*, the promoters of the key nitrogen assimilation genes SpNR and SpNiR exhibited strong G-quadruplex peaks [18]. G-quadruplexes have been found to be non-randomly distributed across the genome, particularly associated with regulatory regions of tobacco [19], wheat [20], barley [21], and pea [22]. Additionally, G-quadruplexes showed distinct DNA methylation patterns between transposon genes and non-transposon genes [17]. The specific distribution of G-quadruplexes in plant genomes suggested underlying correlation with gene expression and protein translation [23,24].

The function of G-quadruplex structures in plant development and stress resistance has been demonstrated. In *A. thaliana*, G-quadruplexes were overrepresented in genes encoding proteins with enzyme activity [16]. The G-quadruplexes in the 5′ UTR of SMXL4/5 are directly bound and induced by the zinc-finger protein JUL, which suppresses the translation of SMXL4/5 and restricts phloem differentiation [25]. Genetic and biochemical analyses have demonstrated that RNA G-quadruplex structures play a role in regulating gene expression and plant development [26]. A study in rice has demonstrated that G-quadruplexes exhibit location-dependent effects on gene expression, with promoter-localized G-quadruplexes positively correlating with expression and gene body-localized ones showing an inverse correlation [27].

G-quadruplex has been proven to promote the adaptation of plants in cold environments by enhancing the stability of mRNA [28]. In the maize genome, G-quadruplexes were enriched in genes associated with energy status, hypoxia, low sugar, and nutrient deprivation [29]. A study on sorghum suggested that the stress-responsive G-rich motif may be recognized by polyadenylation factors or RNA-binding proteins through the formation of G-quadruplex structures [30]. In *Nicotiana tabacum*, G-quadruplexes were enriched in drought stress-responsive genes and related gene families [19]. The *Arabidopsis* Pif-like helicase 1 mutant led to the redistribution of G-quadruplexes in the genome, with differentially enriched peaks affecting genes related to salt stress and heat stress [31].

Grapevine is a nutrient-rich fruit and an important economic crop, and it holds significant value in plant studies [32]. However, as a crucial regulatory element, the specific roles of G-quadruplex structures in grapevine stress tolerance, berry development, and quality formation have not yet been fully elucidated. The progressive improvement of genome assembly quality and the rapid advancement of bioinformatics have provided an opportunity for the study of grape G-quadruplexes [33,34]. This study thoroughly analyzed the distribution characteristics and potential functions of grapevine G-quadruplexes using T2T genome and transcriptome data, including genomic landscape, feature region distribution, G-quadruplexes associated with drought-responsive genes, and G-quadruplexes linked to key genes in sugar–acid metabolism. The findings significantly enhanced the understanding of the prevalence and function of G-quadruplexes in grapevine and horticultural plants.

## 2. Results

### 2.1. General Landscape of G-Quadruplexes in Vitis vinifera Genome

A total of 467,813 G-quadruplexes were identified in the *V. vinifera* nuclear genome, and the number and density of G-quadruplexes vary among chromosomes (Table 1). Chromosome PN18 formed the highest number of G-quadruplexes (32,786), while chromosome PN3 developed the lowest number (18,839). The G-quadruplex density on grapevine chromosomes ranged from 0.8 (PN16, PN19) to 1.1 (PN8, PN11) per kilobase. The average density of G-quadruplex in the grapevine genome was 0.95 occurrences per 1000 base pairs. A significant difference in G-quadruplex formation was observed in the centromeres (Table S1). In the centromere of PN19, only one G-quadruplex was detected. In the centromere of PN15, the 2883 G-quadruplexes were determined, with a G-quadruplex density of 1.61 per kbp. The 105 and 1092 G-quadruplexes were formed in the chloroplast and mitochondrial genomes, with densities of 0.72 and 1.41, respectively.

**Figure 1:**
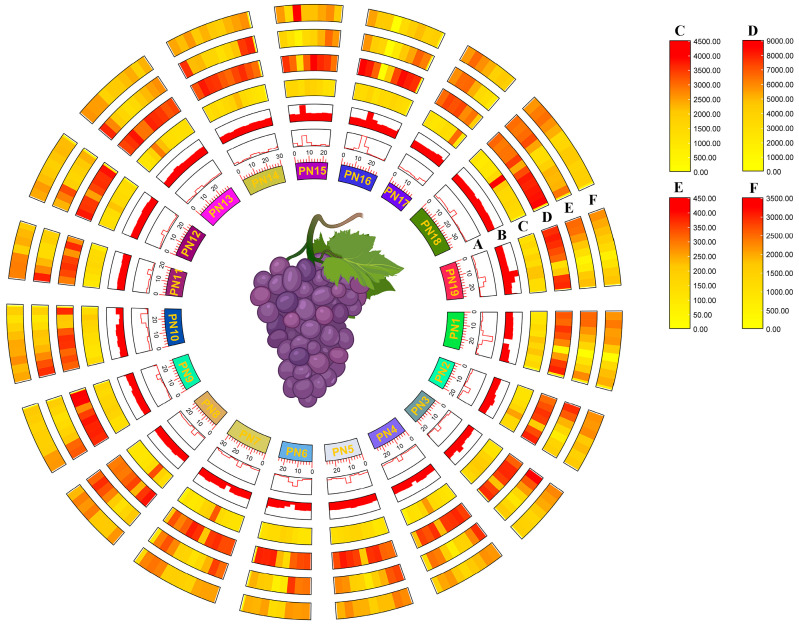
G-quadruplex landscape in grapevine genome. (A) GC skew. (B) GC content. (C) Gene density. (D) Tandem repeats density. (E) TE density. (F) G-quadruplex density.

**Figure 2:**
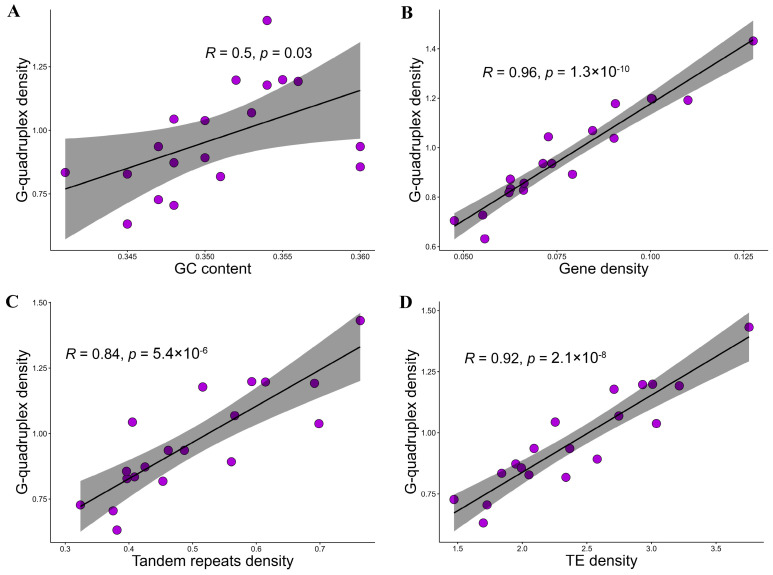
The correlation between G-quadruplexes and genomic characteristics of (**A**) GC content, (**B**) gene density, (**C**) tandem repeat density, and (**D**) TE density at the chromosome level. The gene density, tandem repeat density, and TE density represented the number per 1000 base pairs on the chromosome.

The genome-wide G-quadruplex landscape was presented, allowing the uncovering of mutual influence between G-quadruplexes and genomic features (Figure 1). To clarify the potential relationships between G-quadruplexes and genomic features, the correlation between G-quadruplex density and four genomic features, including GC content, gene density, tandem repeat density, and transposable element density, were investigated at the chromosome level. The results showed that G-quadruplexes were consistently and significantly positively correlated with all four genomic features (Figure 2).

### 2.2. Distribution Patterns of G-Quadruplexes in the Genomic Feature Regions

A large number of G-quadruplexes were characterized in each feature region (Table S2). The 272,652, 81,450, and 37,562 G-quadruplexes were characterized in gene region, promoter2000 region, and TSS500 region, respectively. G-quadruplexes were also frequently found in other feature regions, including exons, CDS, introns, 5′ UTR, and 3′ UTR.

**Figure 3:**
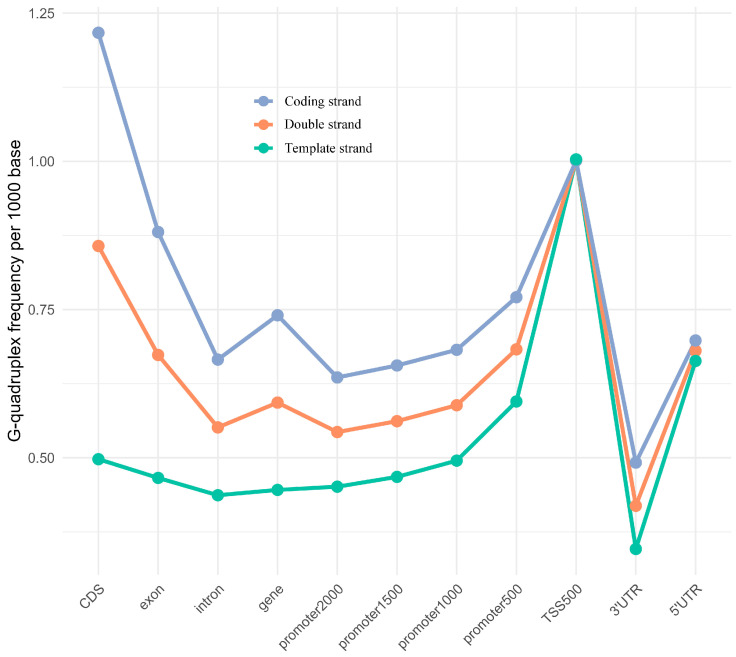
The G-quadruplex density in different feature regions of grapevine genome. The promoter2000, promoter1500, promoter1000, and promoter500 represent the sequences located 2000 bp, 1500 bp, 1000 bp, and 500 bp upstream of the gene, respectively. TSS500 represents the sequence composed of 250 bp on each side of the transcription start site.

Different G-quadruplex densities were found across these feature regions, and differences were also revealed between the template strand and the coding strand (Figure 3). The G-quadruplex density in the TSS500 region was 1.00/kbp, the highest among all investigated feature regions. The G-quadruplex density in the promoter2000 region was also relatively high, at 0.54/kbp. For the promoter region, as the truncated promoter sequences became shorter, the G-quadruplex density gradually increased. For the remaining feature regions, the G-quadruplex density was highest in CDS, followed by 5′ UTR, exon, gene, intron, and lowest in 3′ UTR. Almost equal G-quadruplex densities were observed in both the coding and template strands of the TSS500 region. In other feature regions, the G-quadruplex density on the coding strand was consistently higher than that on the template strand. The conserved strand preference pattern of G-quadruplexes in a specific feature region was particularly evident in the CDS region, where the density on the coding strand was 2.45-fold higher than that on the template strand. The highest G-quadruplex density (1.22/kb) of single-stranded regions was exposed on the coding strand in the CDS.

### 2.3. Functions of Genes with Highly Enriched G-Quadruplexes in Promoters and TSSs

**Figure 4:**
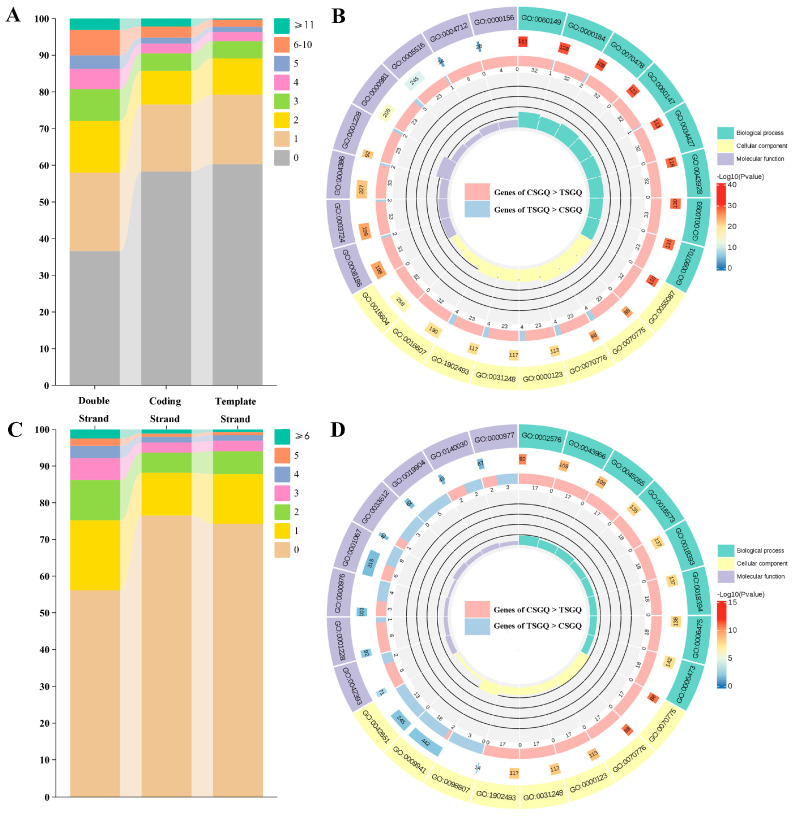
The proportion and GO enrichment of genes involved in G-quadruplexes. (**A**) The proportion of genes with different G-quadruplex numbers in the promoter. (**B**) GO enrichment of genes rich in promoter G-quadruplex (≥11) in double strand. (**C**) The proportion of genes with different G-quadruplex numbers in the TSS500 region. (**D**) GO enrichment of genes rich in TSS500 G-quadruplex (≥6) in double strand. In the GO circle plot, the outermost circle represents the IDs of GO terms. The second circle indicates the total number of genes for each term, and the color of the heatmap represents −log10(Pvalue). The third circle shows the number of enriched genes, with pink/blue boxes representing the number of genes where the G-quadruplex density in the coding strand (CSGQ) was greater than/less than that in the template strand (TSGQ). The fourth circle represents the Rich factor.

G-quadruplexes were enriched in different amounts in the promoters and TSSs of genomic genes, and there were also differences across three sequence contexts (double, coding strand, and template strand) (Figure 4). G-quadruplexes were detected in the promoter2000 regions of 23,755 genes, accounting for 63.39% of all genes in the genome (Table S3). The promoter2000 regions of 1152 genes, representing 3.07% of all genes, contained more than 10 G-quadruplexes (Figure 4A). The 1152 genes were significantly enriched in many GO terms by Gene Ontology (GO) enrichment analysis, indicating their multiple roles in biological processes, cellular components, and molecular functions (Figure 4B). The biological process category included regulation of post-transcriptional gene silencing (GO:0060149, GO:0060147), nuclear-transcribed mRNA catabolic process (GO:0000184, GO:0070478, GO:0034427), and specification of plant organ identity (GO:0010093, GO:0090701). The cellular component category included the H3 histone acetyltransferase complex (GO:0070775), MOZ/MORF histone acetyltransferase complex (GO:0070776), histone acetyltransferase complex (GO:0000123), and protein acetyltransferase complex (GO:0031248). The molecular function category included RNA helicase activity (GO:0003724), helicase activity (GO:0004386), and DNA-binding transcription activator activity-RNA polymerase II-specific (GO:0001228).

G-quadruplexes were found in the TSS500 regions of 16,435 genes, accounting for 43.85% of all genes in the genome (Table S4). The 936 genes existed with more than 5 G-quadruplexes in their TSS500 regions, accounting for 2.50% of all genes (Figure 4C). The essential functions of these 936 genes were revealed through GO enrichment (Figure 4D). The biological process category included histone H3 acetylation (GO:0043966), histone acetylation (GO:0016573), internal peptidyl-lysine acetylation (GO:0018393), peptidyl-lysine acetylation (GO:0018394), internal protein amino acid acetylation (GO:0006475), and protein acetylation (GO:0006473). The cellular component category included H3 histone acetyltransferase complex (GO:0070775), MOZ/MORF histone acetyltransferase complex (GO:0070776), histone acetyltransferase complex (GO:0000123), and protein acetyltransferase complex (GO:0031248). The molecular function category included DNA-binding transcription activator activity-RNA polymerase II-specific (GO:0001228), transcription cis-regulatory region binding (GO:0000976), transcription regulatory region nucleic acid binding (GO:0001067), protein domain-specific binding (GO:0019904), and RNA polymerase II transcription regulatory region sequence-specific DNA binding (GO:0000977).

### 2.4. Prevalence of G-Quadruplexes in DEGs Under Drought Stress

**Figure 5:**
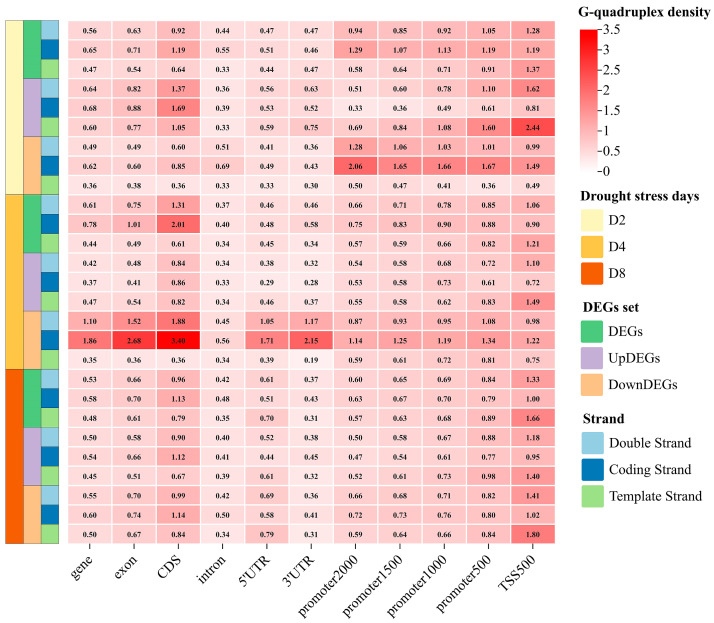
G-quadruplex density of DEGs under drought stress. D2, drought stress for two days; D4, drought stress for four days; D8, drought stress for eight days. UpDEGs, upregulated DEGs; DownDEGs, downregulated DEGs.

A large number of differentially expressed genes were found under drought stress of two days, four days, and eight days (Tables S5–S7). The distinct G-quadruplex distribution was discovered in DEGs under drought stress for two days (Figure 3 and Figure 5). For the G-quadruplex density in the TSS500, promoter2000, promoter1500, promoter1000, and promoter500 regions, DEGs exhibited higher density compared to all genes in the genome. Similarly, for the coding strand G-quadruplex density in these five feature regions, DEGs showed higher densities than the genome-wide genes. However, for the coding strand G-quadruplex density in the remaining feature regions, including gene, exon, CDS, intron, 5′ UTR, and 3′ UTR, DEGs exhibited lower densities than the genome-wide genes (Figure 5). 

**Figure 6:**
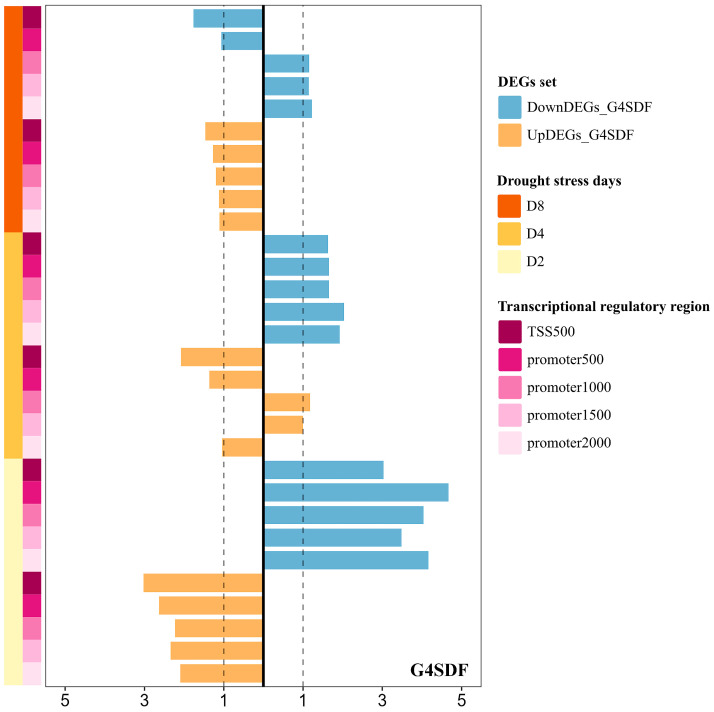
The fold difference in G-quadruplex density between the coding strand and template strand of DEGs under drought stress. The right/left half of the figure represents the fold difference where the coding strand density was greater/less than the template strand density. G4SDF: the fold difference in G-quadruplex density between the two strands.

Notably, opposite G-quadruplex patterns were found in transcriptional regulatory regions of the upregulated DEGs and downregulated DEGs following two days of drought stress, including TSS500, promoter2000, promoter1500, promoter1000, and promoter500 regions (Figure 5). For the G-quadruplex density in these five specific regions, the template strand of upregulated DEGs exhibited higher values than the coding strand, with a fold change ranging from 2.10 to 3.02. In contrast, for downregulated DEGs, the coding strand showed greater values than the template strand, with a fold change ranging from 3.03 to 4.67 (Figure 5 and Figure 6). Extensive significance was also found in these obvious differences (Figure S1).

In the upregulated/downregulated DEGs under drought stress at 4 and 8 days, the fold change in G-quadruplex density between the two complementary strands in the TSS500 and promoter regions (promoter2000, promoter1500, promoter1000, promoter500) gradually decreased compared to the difference of DEGs under drought stress at 2 days (Figure 6). In the downregulated DEGs at 4 days of drought stress, the evidence that the coding strand G-quadruplex density was higher than that of the template strand G-quadruplex density could still be observed, whereas this state was disrupted in the downregulated DEGs at 8 days of drought stress (Figure 6).

**Figure 7:**
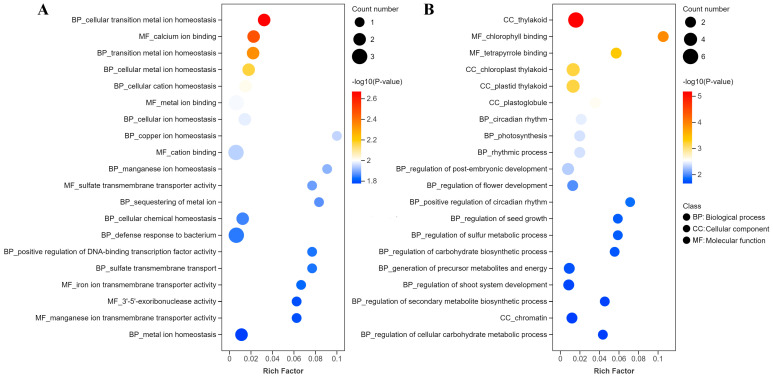
GO function enrichment of representative DEGs under drought stress for two days. (**A**) GO enrichment of upregulated DEGs with a fold change greater than 3 in G-quadruplex density between the coding strand and template strand in the TSS500 region. (**B**) GO enrichment of downregulated DEGs with a fold change greater than 3 in G-quadruplex density between the coding strand and template strand in the TSS500 region.

To further clarify the potential significance of the specific G-quadruplex patterns in DEGs after two days of drought stress, upregulated DEGs and downregulated DEGs with a fold change greater than three in G-quadruplex density between the coding strand and template strand in the TSS500 region were selected as representatives for GO enrichment analysis (Figure 7). The GO enrichment of upregulated DEGs revealed that these genes were broadly associated with multiple metal ion homeostasis in both the biological process and molecular function categories, including cellular transition metal ion homeostasis, cellular cation homeostasis, copper ion homeostasis, manganese ion homeostasis, iron ion transmembrane transporter activity, and the sequestering of metal ions. The biological process of positive regulation of DNA-binding transcription factor activity was also enriched (Figure 7). The GO analysis of downregulated DEGs revealed that these genes were enriched in several biological processes, including circadian rhythm, photosynthesis, regulation of carbohydrate biosynthetic processes, and regulation of cellular carbohydrate metabolic processes (Figure 7).

### 2.5. G-Quadruplexes in the Grape Berry Sugar–Acid Metabolism Pathway

**Figure 8:**
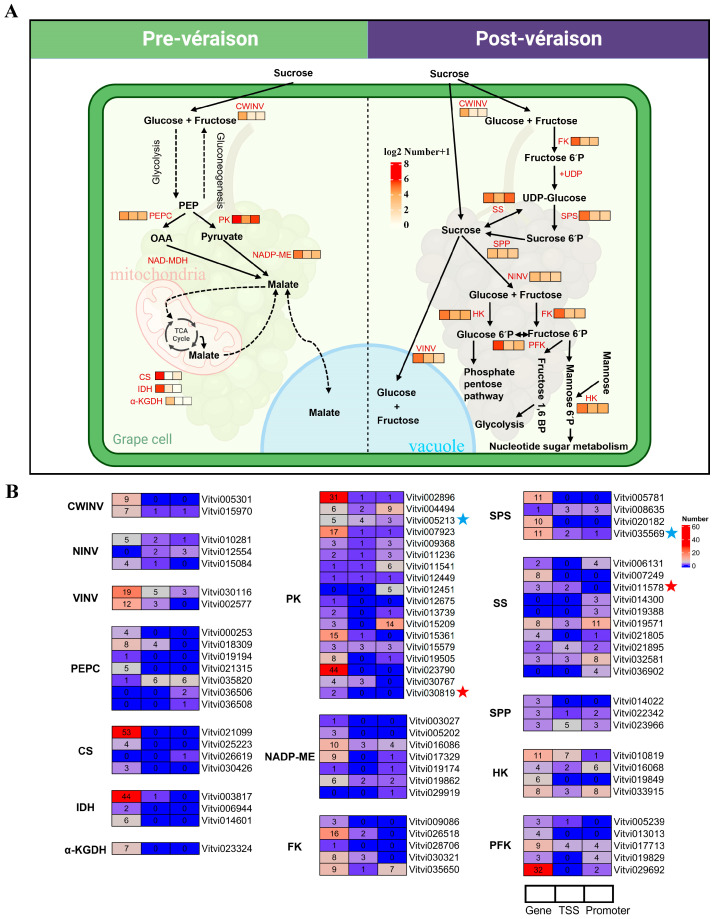
G-quadruplex prevalence of sugar–acid metabolism pathway in the grape berry. (**A**) The sugar–acid metabolic regulation during the pre-véraison and post-véraison stages. (**B**) The G-quadruplex number and differential expression of key genes in the sugar–acid metabolism pathway. The red star and blue star represent significantly upregulated and downregulated gene expression under drought stress, respectively.

The 84 key protease genes, classified into 15 distinct types, were annotated in sugar–acid metabolic pathways, participating in sucrose metabolism, glycolysis, gluconeogenesis, and the tricarboxylic acid cycle (Table S5). The vast majority of these genes contain G-quadruplexes in the gene region, TSS500 region, and promoter region (Figure 8 and Table S5).

The highest number of G-quadruplexes was found in pyruvate kinase (PK) genes, with 148 in the gene region, 19 in the TSS500 region, and 51 in the promoter2000 region (Figure 8). Many G-quadruplexes were also detected in feature regions of other important protease gene categories. For instance, 60, 52, and 51 G-quadruplexes were found in the gene regions of citrate synthase (CS) genes, isocitrate dehydrogenase (IDH) genes, and phosphofructokinase (PFK) genes, respectively. Moreover, 12, 12, and 10 G-quadruplexes were identified in the TSS500 regions of sucrose synthase (SS) genes, hexokinase (HK) genes, and phosphoenolpyruvate carboxylase (PEPC) genes. Additionally, in the promoter2000 regions of SS genes, HK genes, and PFK genes, 36, 15, and 10 G-quadruplexes were detected, respectively (Figure 8). G-quadruplex expansion was particularly prevalent in several genes. A total of 53 G-quadruplexes were identified in the gene region of *Vitvi021099* (CS). In the TSS500 region of *Vitvi010819* (HK), seven G-quadruplexes were found. Additionally, 14 G-quadruplexes were observed in the promoter region of *Vitvi015209* (PK) (Figure 8). The CWINV, NINV, and VINV genes contained G-quadruplexes in different quantities: 16, 9, and 31 in their gene body regions; 1, 5, and 8 within TSS500 regions; and 1, 4, and 3 in promoter regions. Additionally, significant differences in the number and distinct motif patterns of G-quadruplexes between NINV and VINV genes were revealed.

These key protease genes engaged in sugar acid metabolism, which possess G-quadruplexes, also respond to drought stress. The expression of the *Vitvi030819* (PK) was significantly upregulated after two days of drought stress, with two G-quadruplexes in its gene region. The *Vitvi011578* (SS) was significantly upregulated after eight days of drought stress, with three G-quadruplexes present in its gene region and two in the TSS500 region. Furthermore, *Vitvi005213* (PK) and *Vitvi035569* (SPS) showed significant downregulation after eight days of drought stress, and G-quadruplexes were also found in their gene regions, TSS500 regions, and promoter2000 regions. These results suggest that G-quadruplexes might play an extensive regulatory role as gene expression regulators in drought responses and quality-related metabolism.

## 3. Discussion

### 3.1. High-Density G-Quadruplexes May Facilitate Grapevine Adaptive Evolution

G-quadruplexes were widely and diversely distributed in the grapevine genome. The G-quadruplex density in the grapevine nuclear genome was 0.95 per kbp, which was higher than that of herbaceous plants identified by the same means, such as *A. thaliana* (0.34 per kbp) [35], pea (0.42 per kbp) [22], and tobacco (0.5 per kbp) [19]. In grapevine, the G-quadruplex density in the mitochondrial genome was higher than that in the chloroplast genome, suggesting an important regulatory role of G-quadruplexes in mitochondria [36]. G-quadruplex density was confirmed to be significantly positively correlated with TE density, suggesting that TE may play a crucial role in G-quadruplex expansion. The high-density G-quadruplexes as a form of epigenetic regulation may have facilitated environmental adaptation in woody plants such as grapevine [37] and potentially enhanced the cost–benefit ratio of the genome [38]. The impact of G-quadruplex sequence variations mediated by nucleotide polymorphisms on gene expression and local adaptation will be further elucidated with the assembly of the *Vitis* pan-genome [39,40].

Plant centromeres are composed of highly repetitive satellite DNA and retrotransposon elements [41,42], ensuring accurate chromosome segregation and maintaining genomic stability [43,44]. Several studies have uncovered the prevalence of non-B DNA at centromeres [45,46,47,48]. G-quadruplexes were distributed within the long terminal repeat retrotransposons of *Citrus sinensis* centromeres [49]. In the oat centromere, G-quadruplexes formed within the CENH3-binding regions [50]. Benefiting from the complete genome of grapevine, we found that G-quadruplexes were unevenly distributed among all centromeres. The centromere of chromosome PN15 exhibited the highest G-quadruplex density (1.61/kbp), which may have contributed to enhancing the structural stability of this region, thereby more effectively ensuring the accurate transmission of genetic information. Additionally, the presence of high-density G-quadruplexes in PN15 might influence chromatin accessibility, thereby regulating gene transcription activity. However, the lower G-quadruplex density in other centromeres of grapevine suggested that genomic regulation in these regions might rely on alternative mechanisms, such as histone modifications or DNA methylation [51]. In the future, the causes and consequences of the significant differences in G-quadruplex formation among different centromeres would be an interesting question. The genomic structural heterogeneity might have represented different adaptive evolutionary patterns of grape centromeres among different chromosomes.

### 3.2. G-Quadruplexes Enriched in Transcriptional Regulatory Regions with Strand Bias

In the grapevine promoter and TSS500 regions, 81,450 and 37,562 G-quadruplexes were identified, respectively. As the transcription start site was approached, the density of G-quadruplexes increased. In these two regulatory regions, the G-quadruplex densities were 0.54/kbp and 1.00/kbp, respectively, showing higher G-quadruplex density compared to tobacco [19]. Notably, in these transcriptional control regions of the grapevine genome, the G-quadruplex density on the coding strand was greater than that on the template strand.

Genes with G-quadruplexes enriched in genetic regulatory regions were defined as genes with more than ten G-quadruplexes in the promoter and genes with more than five G-quadruplexes in TSS500. GO enrichment analysis indicated that these genes were involved in important biological processes in grapevine, including post-transcriptional gene silencing (GO:0060149, GO:0060147), histone acetylation (GO:0016573, GO:0043966), and specific binding of the transcription regulatory region (GO:0000976, GO:0001067). In rice, K^+^-specific G-quadruplexes were correlated with active histone marks and low DNA methylation levels [52]. It is worth noting that genes with the same GO function (e.g., GO0060149, GO0008186, GO0002576, GO0019904) almost all exhibited the same strand preference pattern for their G-quadruplexes. In the human genome, G-quadruplexes were enriched in the promoter regions as cis-acting elements, and their functions and therapeutic potential have garnered widespread attention [5,53,54]. The significant enrichment of G-quadruplexes in the transcriptional regulatory regions, along with conserved strand preference distribution, underscores their indispensable function in the regulation of gene expression in grapevine.

### 3.3. G-Quadruplex Might Participate in the Rapid Response to Drought Stress in Grapevine

To uncover the potential role of G-quadruplexes in abiotic stress, G-quadruplexes of DEGs under drought stress were specifically investigated. Using time-series transcriptomic data, the unique G-quadruplex pattern in DEGs under two days of drought stress was illustrated. In the promoter and TSS500 regions, the G-quadruplex density of DEGs was higher than that of all genes in the genome. The high G-quadruplex density phenotype in transcriptional regulatory regions was also observed in the DEGs under drought stress in *A. thaliana* and tobacco [19,35]. It was noteworthy that the opposite strand preference in the G-quadruplex distribution of different categories of DEGs was uncovered. For upregulated DEGs, the G-quadruplex density on the template strand was higher than that on the coding strand in the promoter and TSS500 regions. For downregulated DEGs, the density on the coding strand was greater than that on the template strand. Several studies have confirmed the impact of strand positioning on the function of G-quadruplexes. For example, the insertion of G-quadruplex sequences in the upstream regulatory region of the *Escherichia coli* expression system significantly enhanced transcription and translation efficiency when G-quadruplex structures formed in the coding strand. However, both transcription efficiency and fluorescence intensity were reduced when G-quadruplex sequences were inserted in the template strand [55]. The fold difference of G-quadruplex density in grapevine DEGs under drought stress between the two strands was around three, representing a conserved and undeniable distinction.

The upregulated genes with G-quadruplex strand preference were associated with the transport and homeostasis of various metal ions. The regulation of metal ions involved important processes such as osmotic regulation, the activation of antioxidant defense systems, and the protection of the photosynthetic system under drought stress [56,57,58]. Based on the ability of metal ions to stabilize G-quadruplex structures, we hypothesized that G-quadruplexes may function as receptor elements, allowing plants to rapidly respond to environmental changes without the need to synthesize new proteins; thus, this might help plants conserve valuable energy during drought. A similar but weaker high G-quadruplex density pattern to that observed in the second-day DEGs was also found in the DEGs on the fourth and eighth days of drought stress. These results emphasized that G-quadruplexes enriched in transcriptional regulatory regions may widely regulate drought stress tolerance and play a key role in rapid stress response. The molecular mechanisms by which G-quadruplexes participate in drought stress responses in grapevine can be further investigated through the integration of biophysical and molecular biological approaches [59,60].

Extensive and significant G-quadruplex differences were also identified among DEGs under 2-day, 4-day, and 8-day drought stress conditions. While no statistical significance was detected in several gene sets, we observed approximately threefold differences between the template and coding strands, representing a pronounced differential trend. In fact, the chain localization of G-quadruplex has been found to play a key role in biological processes. For example, recent scientific research shows that the DNA unwinding activity mediated by the Cdc45-MCM-GINS helicase complex often encounters structural barriers within chromosomal DNA, such as G-quadruplex structures, that may induce replication stress and compromise genomic integrity [61].

### 3.4. G-Quadruplexes as Targets of Sugar–Acid Regulation of Grape Berry

Sugar and acid metabolism was a core physiological process in grape berry development and stress response, and its dynamic balance directly affected fruit quality, flavor formation, and plant stress resistance [60,62,63]. G-quadruplexes were abundant in the key genes associated with sucrose metabolism, glycolysis, gluconeogenesis, and the tricarboxylic acid cycle in grapevine. In the TSS500 regions of SS, HK, and PK genes, there were 12, 12, and 19 G-quadruplexes, respectively, while in the promoter regions, there were 36, 15, and 51 G-quadruplexes, respectively. Multiple G-quadruplexes were also distributed in CS, IDH, PFK, and PEPC genes. In maize, G-quadruplexes were found to be present in genes associated with sugar metabolism [29]. In the grapevine genome, the number of G-quadruplexes in the VINV gene was higher than that in CWINV and NINV, particularly in the gene body region. The differences in G-quadruplex distribution among the three types of sucrose transferases may be attributed to variations in the GC content of their nucleotide sequences and could be related to their unique subcellular localization and functional specificity [64].

GO enrichment analysis revealed that genes involved in the regulation of carbohydrate biosynthesis and metabolic processes participated in drought stress resistance. The PK gene *Vitvi030819* and the SS gene *Vitvi011578* were significantly upregulated under drought stress, with two and three G-quadruplexes identified in their gene body regions, respectively. G-quadruplexes are expected to serve as important epigenetic targets for the precise regulation of sugar and acid metabolism based on the CRISPR/Cas system in the future, aiming to synergistically optimize berry flavor and stress adaptability [65,66]. Notably, biological large language models have recently demonstrated unique advantages in deciphering the relationship between nucleic acid sequences and gene expression regulation [67,68], including the deciphering of G-quadruplexes by PlantRNA-FM [69]. These advancements will provide new strategies and tools for G-quadruplex-based fruit quality improvement and stress-resistant molecular breeding while simultaneously accelerating the establishment of a molecular breeding paradigm based on nucleic acid secondary structure regulation.

## 4. Materials and Methods

### 4.1. Identification of G-Quadruplexes in the Vitis vinifera Genome

The *Vitis vinifera* T2T genome assembly (nucleus, mitochondria, chloroplast) and annotation (gene, tandem repeats, and TE) were retrieved from the Zenodo database (https://zenodo.org/records/10969867 (accessed on 10 September 2024)) [70]. The centromeric sequences were extracted based on the confirmed chromosome location using the SeqKit2 [71]. The G-quadruplexes in chromosomes and centromeres were identified using G4Hunter, with window 25 and threshold 1.2 [72]. The G4Hunter analysis outcomes were further cleaned. Finally, the genomic coordinates of each G-quadruplex were obtained. The G-quadruplex number, G-quadruplex density, sequence length, and GC content in each chromosome and centromere, along with detailed information on all G-quadruplexes, were summarized. The grapevine genome landscape was calculated and displayed using Advanced Circos [73], including G-quadruplex density, GC density, GC skew, gene density, tandem repeat density, and TE density. The correlation analyses were performed using the Pearson test.

### 4.2. G-Quadruplex Analysis of Genomic Feature Regions in the Grapevine Genome

The genome coordinates of feature regions, including exon, CDS, intron, 5′ UTR, 3′ UTR, gene, promoter2000, promoter1500, promoter1000, promoter500, and TSS500, were extracted and calculated based on the genome annotation file. The promoter2000, promoter1500, promoter1000, and promoter500 regions were defined as the sequences located 2000 bp, 1500 bp, 1000 bp, and 500 bp upstream of the gene, respectively. TSS500 was delimited as the sequence composed of 250 bp on each side of the transcription start site. Derived from the genomic coordinates of G-quadruplexes and feature regions, the numbers of G-quadruplexes in these feature regions were quantified, and G-quadruplex densities were further calculated.

### 4.3. Functional Enrichment Analysis of Grapevine G-Quadruplex-Rich Genes

In accordance with the number of G-quadruplexes of the promoter region and TSS500 region in the DNA double strand, template strand, and coding strand, all genes in the grapevine genome were classified, and their percentages were calculated. GO enrichment analysis was performed on two gene clusters: (1) genes with more than 10 G-quadruplexes in the promoter double strand and (2) genes with more than 5 G-quadruplexes in the TSS500 double strand. The functional annotation of all genomic genes was conducted using eggNOG-mapper (http://eggnog-mapper.embl.de/ (accessed on 6 November 2024)) [74]. GO enrichment analysis and visualization were performed by deploying the clusterProfiler 4.6.0 R package [75] based on the Metware Cloud platform (https://cloud.metware.cn (accessed on 8 November 2024)).

### 4.4. G-Quadruplex Analysis of DEGs Under Drought Stress at Different Stages

To probe the G-quadruplex patterns of DEGs under drought stress at various stages, RNA-seq data (SRP132681) from leaf samples collected after 2, 4, and 8 days of drought stress were retrieved from the Sequence Read Archive (SRA) database [76]. The raw sequencing data were assessed for quality through FastQC v0.12.1. Adapter sequences were removed, and the first 12 bases of reads were trimmed to obtain clean reads using Trimmomatic v0.39 [77]. The grapevine genome index was constructed, and clean reads were mapped to the reference genome by HISAT2 v2.2.1 [78]. SAM files were then converted to reordered BAM files via SAMtools v1.7 [79]. Transcript abundances were estimated, and FPKM values were calculated using StringTie v2.1.7 [80]. Gene count values were obtained by the prepDE.py3 script provided by StringTie. DEGs were identified with DESeq2 [81], adopting a |log2FoldChange| ≥ 1 and padj ≤ 0.05 as the threshold.

All DEGs were classified into upregulated DEGs and downregulated DEGs, and the G-quadruplex density in feature regions of these DEGs was calculated. G-quadruplex density was displayed using ChiPlot (https://www.chiplot.online/ (accessed on 2 February 2025)). The upregulated and downregulated DEGs after 2 days of drought stress, with fold change greater than three for G-quadruplex density in template and coding strands of the TSS500 region, were selected for GO enrichment analysis using the clusterProfiler 4.6.0 R package.

### 4.5. G-Quadruplex Analysis of Key Genes in the Sugar–Acid Metabolism Pathway of Grape Berry

The key protease genes involved in the important sugar–acid metabolic pathways of grape berries were gathered from previously published studies [82,83]. The KO Entry of each protease gene was retrieved through the KEGG database [84]. Based on KO Entry and grapevine protein annotations from eggNOG, the key proteins involved in grape sugar–acid metabolism were extracted. The subcellular localization of the proteins was predicted using DeepLoc-2.0 [85]. The number of G-quadruplexes in the gene body, promoter2000, and TSS500 regions of genes encoding these proteins was calculated based on their genomic coordinates. G-quadruplex sequences were aligned using MAFFT v7.525 [86] and then trimmed using trimAl v1.5.0 [87]. The SeqLogo was shown using TBtools v2.210.

## 5. Conclusions

This study revealed that G-quadruplexes are widespread and functionally significant in the grapevine genome. Hundreds of thousands of G-quadruplexes were identified, exhibiting significant enrichment in transcriptional regulatory regions and demonstrating positive correlations with GC content, gene density, and transposon density. The G-quadruplex density within centromeres displays heterogeneity. Under drought stress, differentially expressed genes (DEGs) exhibited distinct strand-biased G-quadruplex patterns. The upregulated DEGs showed template strand dominance in promoters and TSS500 linked to metal ion homeostasis, while downregulated DEGs displayed coding strand preference associated with sugar–acid metabolism. Additionally, G-quadruplexes were highly enriched in key sugar–acid metabolism genes. Sucrose transferases with three distinct subcellular localizations exhibited varying abundances of G-quadruplexes. These findings highlight G-quadruplexes as versatile regulators of grapevine stress adaptation and berry development, offering novel targets for epigenetic engineering to enhance plant resilience and fruit quality.

## Figures and Tables

**Table 1:** G-quadruplex profile of *Vitis vinifera* genome. The G-quadruplexes were identified by G4Hunter, with a window size of 25 and a threshold value of 1.2.

Chromosome	Length (bp)	GC Content (%)	G-Quadruplex Number	G-Quadruplex Density (per kbp)
PN1	27,822,162	36.0	26,041	0.94
PN2	20,941,263	35.2	21,555	1.03
PN3	21,317,290	34.8	18,839	0.88
PN4	25,934,928	34.7	25,232	0.97
PN5	26,899,771	35.3	27,761	1.03
PN6	24,571,969	35.5	23,639	0.96
PN7	31,654,362	34.8	28,552	0.90
PN8	23,763,023	35.0	26,679	1.12
PN9	24,372,199	34.1	22,262	0.91
PN10	27,504,061	35.6	25,068	0.91
PN11	20,048,508	35.0	22,253	1.11
PN12	24,706,008	34.8	24,271	0.98
PN13	29,842,242	34.5	28,749	0.96
PN14	30,475,315	34.5	29,451	0.97
PN15	23,565,456	35.4	22,321	0.95
PN16	27,608,946	36.0	21,207	0.77
PN17	19,942,836	35.4	20,337	1.02
PN18	36,684,271	34.7	32,786	0.89
PN19	27,218,600	35.1	20,810	0.76
mitochondrion	774,663	44.1	1092	1.41
chloroplast	145,134	36.7	105	0.72

## Data Availability

Data are contained within the article or Supplementary Materials.

## Supplementary Materials

The following supporting information can be downloaded at: https://www.mdpi.com/article/10.3390/plants14081180/s1, Figure S1: The difference of G-quadruplex density (/kbp) between coding strand and template strand of differentially expressed genes under drought stress. (A) The density difference of G-quadruplex in promoter 2000, promoter 1500, promoter 1000, promoter 500 and TSS 500 regions of differentially expressed genes was up-regulated after two days of drought stress. (B) The density difference of G-quadruplex in promoter 2000, promoter 1500, promoter 1000, promoter 500 and TSS 500 regions of differentially expressed genes was down-regulated after two days of drought stress. (C) The density difference of G-quadruplex in promoter 2000, promoter 1500, promoter 1000, promoter 500 and TSS 500 regions of differentially expressed genes was up-regulated after four days of drought stress. (D) The density difference of G-quadruplex in promoter 2000, promoter 1500, promoter 1000, promoter 500 and TSS 500 regions of differentially expressed genes was down-regulated after four days of drought stress. (E) The density difference of G-quadruplex in promoter 2000, promoter 1500, promoter 1000, promoter 500 and TSS 500 regions of differentially expressed genes was up-regulated after eight days of drought stress. (F) The density difference of G-quadruplex in promoter 2000, promoter 1500, promoter 1000, promoter 500 and TSS 500 regions of differentially expressed genes was down-regulated after eight days of drought stress. Figure S2: Differences in the number of G-quadruplexes and specific motif patterns in the gene body regions of sucrose transferases. (A) Differences in the number of G-quadruplexes among three sucrose transferases. (B) Specific motif patterns of G-quadruplexes in the NINV genes. (C) Specific motif patterns of G-quadruplexes in the VINV genes. Table S1: G-quadruplex profile of *Vitis vinifera* centromeres; Table S2: The number of G-quadruplexes in feature regions of grapevine genome; Table S3: The number and percentage of genes with different numbers of G-quadruplexes in promoter2000; Table S4: The number and percentage of genes with different numbers of G-quadruplexes in TSS500; Table S5: Differentially expressed genes in two days under drought stress; Table S6: Differentially expressed genes in four days under drought stress; Table S7: Differentially expressed genes in eight days under drought stress; Table S8: The key genes in the sugar–acid metabolic pathway and their G-quadruplex enrichment.
