# Supplementary material for: Decoding G-Quadruplexes Sequence in Vitis vinifera: Regulatory Region Enrichment, Drought Stress Adaptation, and Sugar–Acid Metabolism Modulation
Source: Plants (Basel). 2025 Apr 10;14(8):1180. doi: 10.3390/plants14081180 (PMC12030360; doi:10.3390/plants14081180)
Supplement: Supplementary file 1 [file plants-14-01180-s001.zip › Supplementary Materials.pdf]

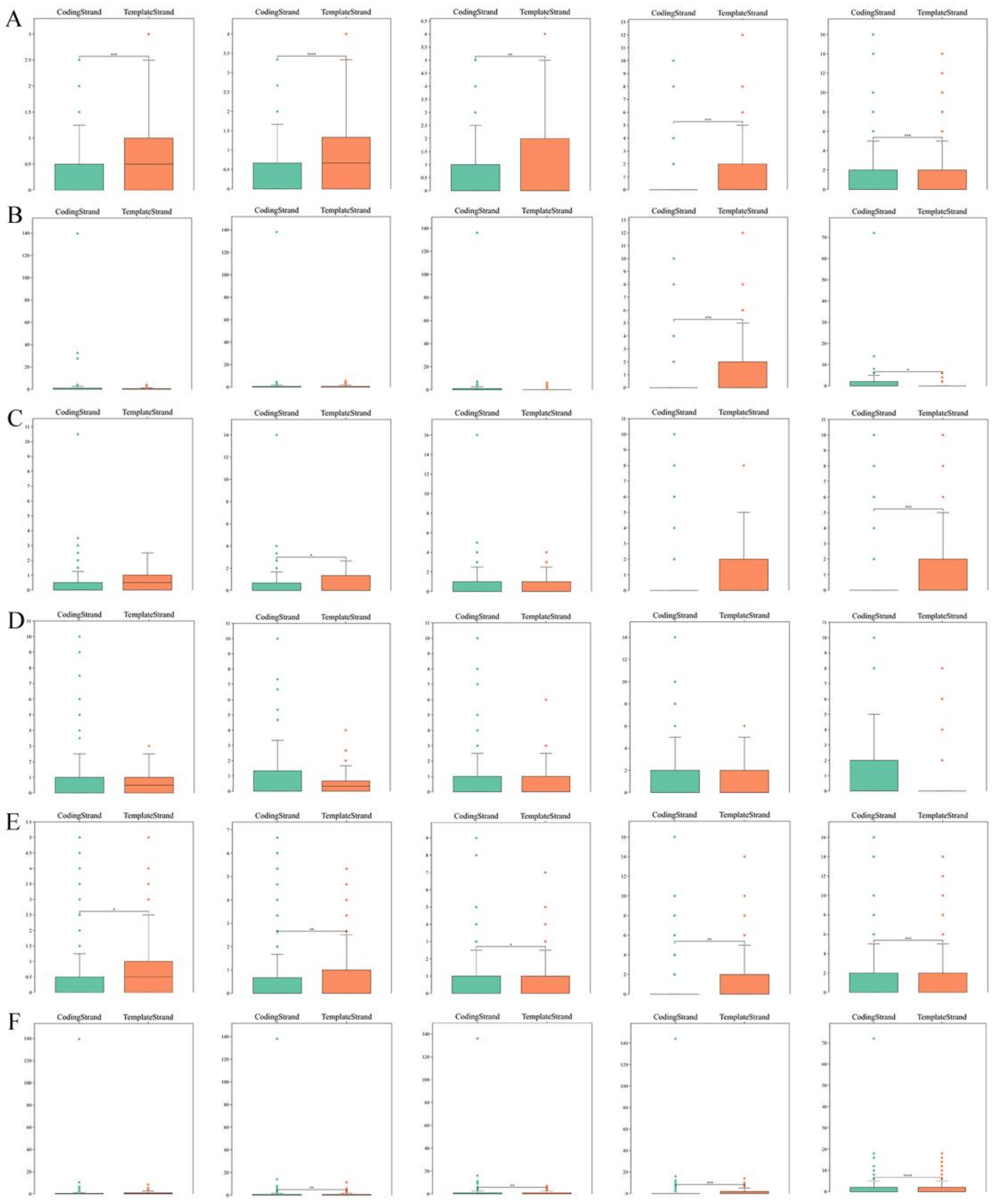

Figure S1. The difference of G-quadruplex density (/kbp) between coding strand and template strand of differentially expressed genes under drought stress. (**A**) The density difference of G-quadruplex in promoter 2000, promoter 1500, promoter 1000, promoter 500 and TSS 500 regions of differentially expressed genes was up-regulated after two days of drought stress. (**B**) The density difference of G-quadruplex in promoter 2000, promoter 1500, promoter 1000, promoter 500 and TSS 500 regions of differentially expressed genes was down-regulated after two days of drought stress. (**C**) The density difference of G-quadruplex in promoter 2000, promoter 1500, promoter 1000, promoter 500 and TSS 500 regions of differentially expressed genes was up-regulated after four days of drought stress. (**D**) The density difference of G-quadruplex in promoter 2000, promoter 1500, promoter 1000, promoter 500 and TSS 500 regions of differentially expressed genes was down-regulated after four days of drought stress. (**E**) The density difference of G-quadruplex in promoter 2000, promoter 1500, promoter 1000, promoter

500 and TSS 500 regions of differentially expressed genes was up-regulated after eight days of drought stress. (**F**) The density difference of G-quadruplex in promoter 2000, promoter 1500, promoter 1000, promoter 500 and TSS 500 regions of differentially expressed genes was down-regulated after eight days of drought stress.

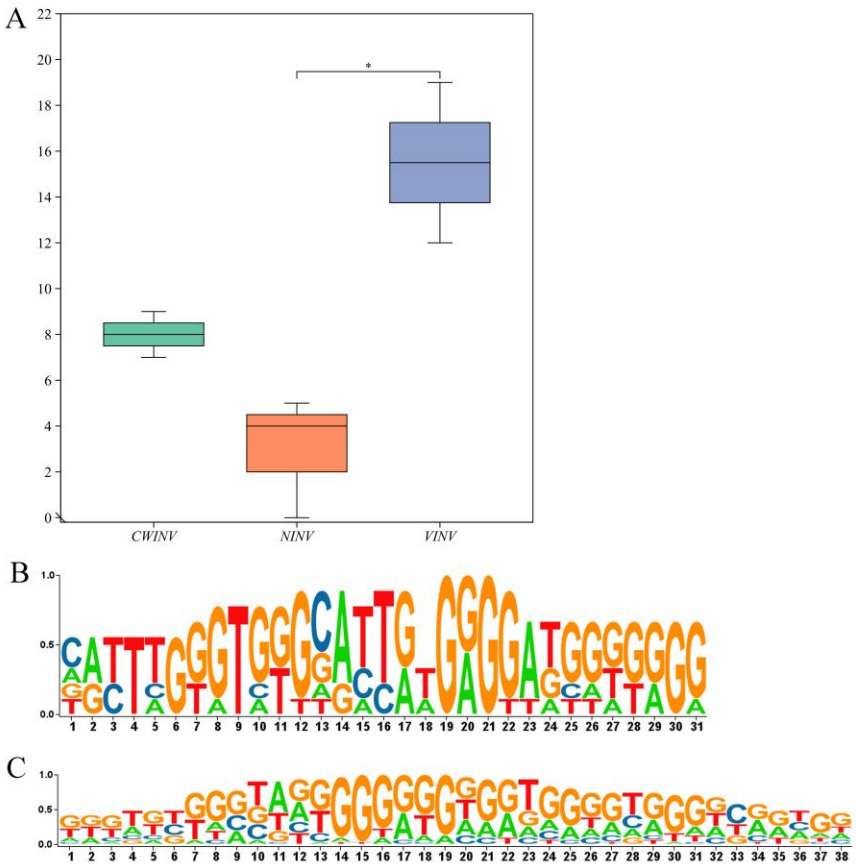

Figure S2. Differences in the number of G-quadruplexes and specific motif patterns in the gene body regions of sucrose transferases. (**A**) Differences in the number of G-quadruplexes among three sucrose transferases. (**B**) Specific motif patterns of G-quadruplexes in the NINV genes. (**C**) Specific motif patterns of G-quadruplexes in the VINV genes.
