# Supplementary figures and images for: Decoding G-Quadruplexes Sequence in Vitis vinifera: Regulatory Region Enrichment, Drought Stress Adaptation, and Sugar–Acid Metabolism Modulation
Source: Plants (Basel). 2025 Apr 10;14(8):1180. doi: 10.3390/plants14081180 (PMC12030360; doi:10.3390/plants14081180)

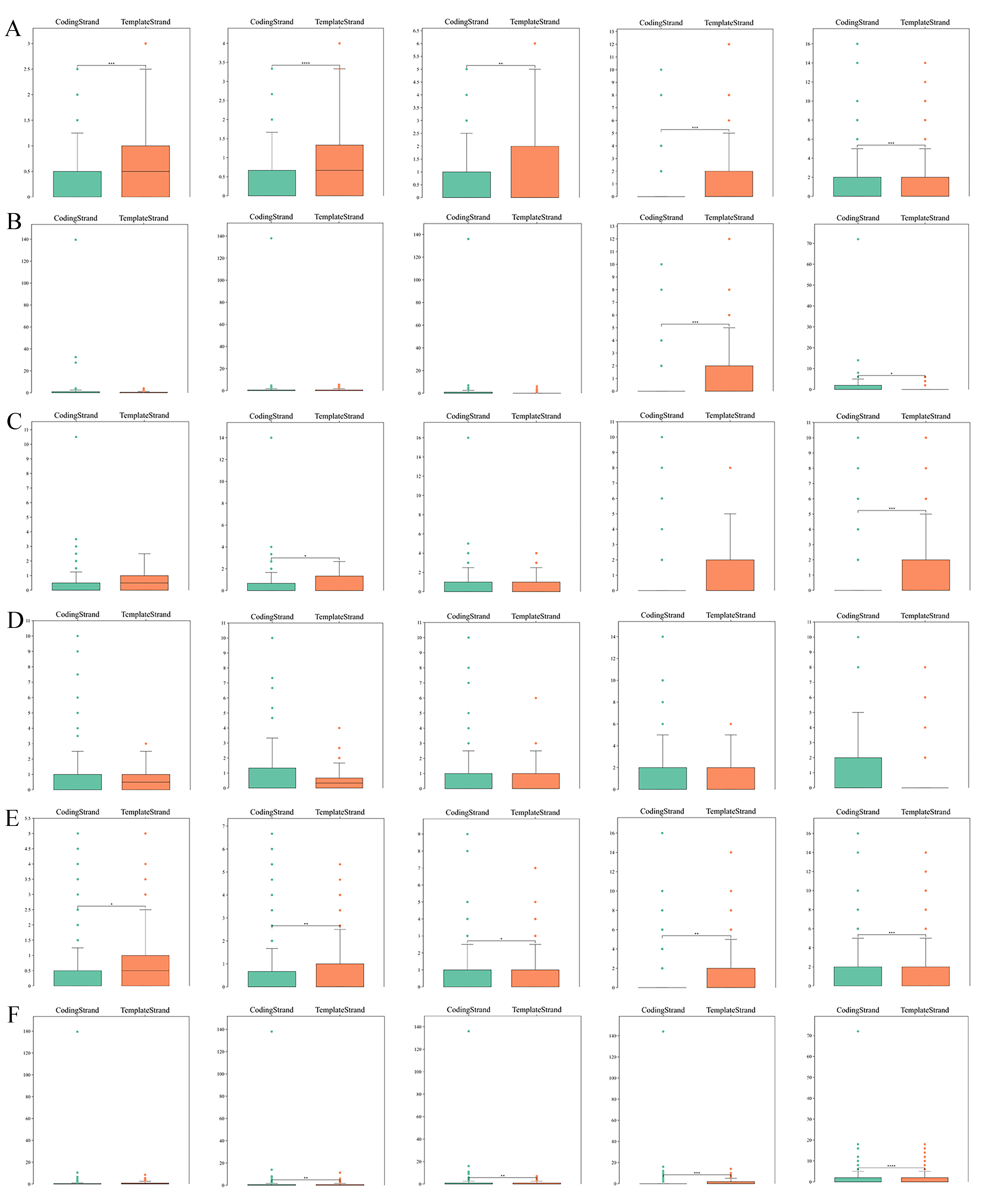

Supplement: Supplementary file 1 [file plants-14-01180-s001.zip › Figure S1.jpg]

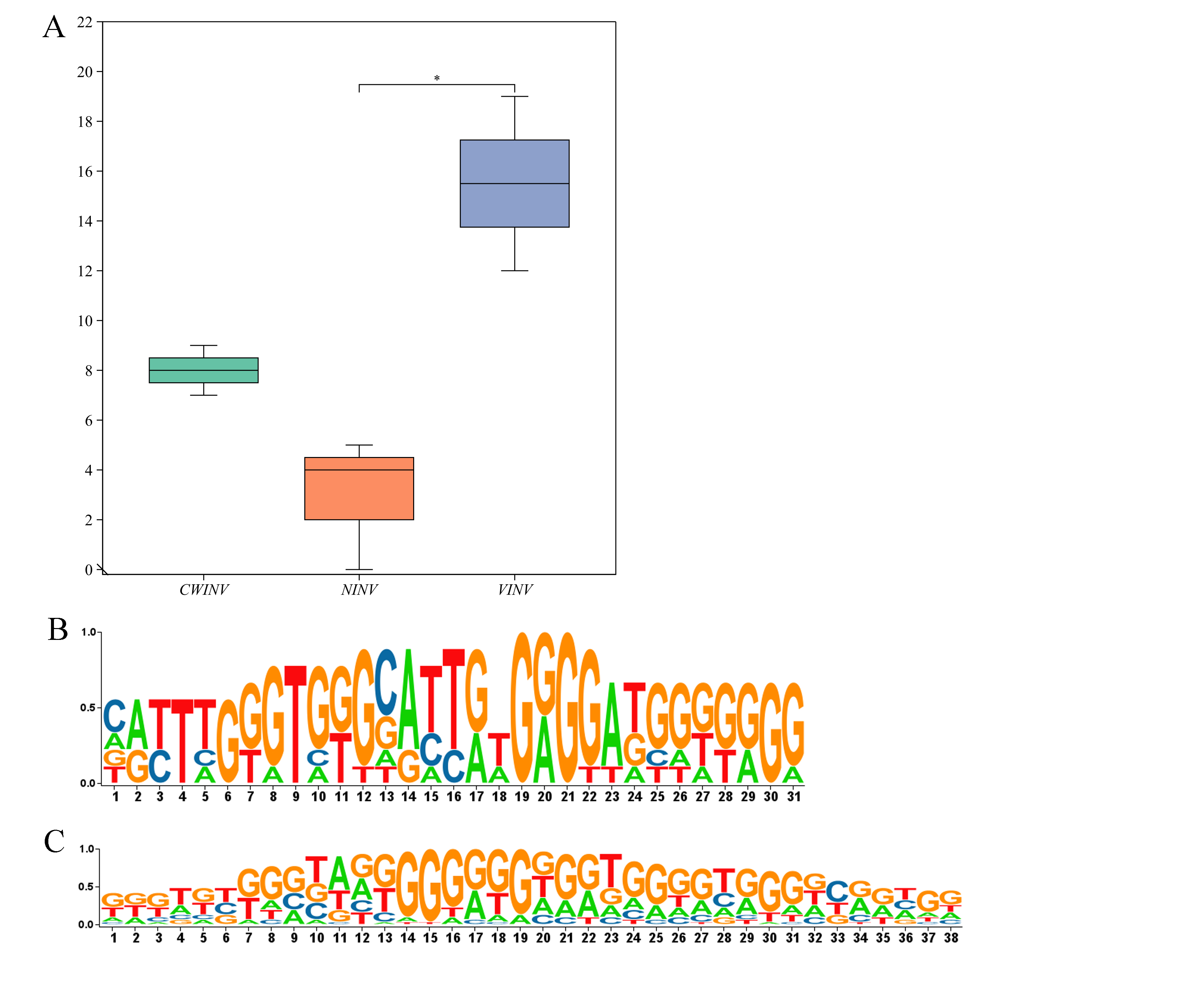

Supplement: Supplementary file 1 [file plants-14-01180-s001.zip › Figure S2.jpg]
